# Higher Ki67 expression in fibroblast like cells at invasive front indicates better clinical outcomes in oral squamous cell carcinoma patients

**DOI:** 10.1042/BSR20181271

**Published:** 2018-11-21

**Authors:** Yue Jing, Yan Yang, Fengyao Hao, Yuxian Song, Xiaoxin Zhang, Ye Zhang, Xiaofeng Huang, Qingang Hu, Yanhong Ni

**Affiliations:** 1 Central Laboratory, Nanjing Stomatological Hospital, Medical School of Nanjing University, Nanjing, China; 2 Department of Oral and Maxillofacial Surgery, Nanjing Stomatological Hospital, Medical School of Nanjing University, Nanjing, China

**Keywords:** fibroblast like cells, immunohistochemistry, Ki67, OSCC, prognostic marker, tumor cells

## Abstract

**Background**: Ki67 has been a key role for the treatment options and prognosis evaluation in some kinds of tumors; however, the spatial expression of Ki67 in oral squamous cell carcinoma (OSCC) has not been fully-evaluated. Therefore, in the present study, we aimed to elucidate the prognosis value of Ki67 spatial expression including in different cell types and at different compartments of tumor in OSCC patients. **Methods:** Immunohistochemical expression of Ki67 in tumor cells (TCs) and fibroblast like cells (FLCs) at center of tumor (CT) and invasive front (IF) was evaluated in 109 OSCC patients. Then correlations of Ki67 expressions with clinicopathological parameters were analyzed by Chi-square test, and survival curves were evaluated by Kaplan–Meier methods. Furthermore, univariate and multivariate analysis were performed to assess the diagnostic values of Ki67 expression by the Cox regression model. **Results:** Ki67 expression in TCs was much higher than in FLCs both at CT and IF compartments, but Ki67 expression in TCs was simultaneously higher at CT than that at IF (*P*=0.0004), which was converse to Ki67 expression in FLCs (*P<*0.0001). Additionally, high Ki67 expression in FLCs at IF was significantly associated with poor tumor differentiation (*P*=0.003), worse depth of invasion (DOI, *P*=0.027) and worst pattern of invasion (WPOI, *P*=0.041), but Ki67 expression in TCs had no correlation with clinical parameters no matter at CT or IF. Moreover, patients with higher Ki67 expression in TCs at CT had significantly increased risk for OS (overall survival; HR:1.935, 95% CI: 1.181–4.823, *P*=0.0395) and DFS (disease-free survival; HR: 2.974, 95% CI:1.189–5.023, *P*=0.046). On contrary, higher Ki67 expression in FLCs at IF was correlated with better OS (HR: 0.15, 95% CI: 0.018–0.846, *P*=0.0396) and DFS (HR: 0.15, 95% CI: 0.018–0.947, *P*=0.0445). Whereas, Ki67 expression both at TCs in IF and at FLCs in CT had no significant prognostic value for OS and DFS. Furthermore, Cox multivariate analysis revealed that Ki67 expression in FLCs at IF could not be an independent prognostic factor for OSCC patients. **Conclusion:** These results show that higher Ki67 expression in FLCs at IF indicated better clinical outcomes for OSCC patients.

## Introduction

Oral squamous cell carcinoma (OSCC) ranks the most common cancer of the oral cavity [1], which has different levels of tumor differentiation, and is inclined to metastasize to the lymph nodes [2]. These lead to treatment failures and disappointed 60% 5-year survival rate [3]. Since OSCC development is a complicated process and limited available knowledge on factors promoting oncogenic transformation to malignant cells is known, it is challenging to explore valuable factors involved in its progression [4].

Ki67, also known as Ki67 antigen or MKI67 (marker of proliferation Ki67), has been a well-established marker for predicting the clinical outcomes for patients with several types of cancer [5–8]. Previous data indicated that a tumor expressed higher Ki67 carried a poor prognosis [9–11]. In addition to conventional parameters, Ki67 has been proposed as a key factor in making tumor treatment decision [12].

With the progress of new techniques, researches have revealed the potential roles of tumor heterogeneity underlying the mechanism of tumor relapse and metastasis [13]. It is universally recognized that heterogeneity includes spatial differences between distinctive subpopulation of TCs within any individual tumor at both genetic and epigenetic levels [14]. Moreover, tumor environment components including tumor cells (TCs), fibroblast like cells (FLCs), infiltrating immune cells, extracellular matrix, vascular endothelia cells confer specific subsets of cancer cells and interact with cancerous subpopulations [15]. Regarding OSCC, multiple studies have stated its tumor heterogeneity at molecular, histological, and phenotypic levels [16–18]. For instance, our previous data found that distinct expression patterns of TLR7 in TCs and FLCs predicted different clinical outcomes for OSCC patients [19]. Many studies have shown a close relationship between Ki67 index with tumor size, angio-invasion, and some other biological behavior of malignant tumors [20–23]. However, spatial expression of Ki67 in tumors is under debate [24,25].

In the present study, we focused on Ki67 expression in TCs and stroma FLCs particularly, and at different compartments, center of tumor (CT) and invasive front (IF). Next, we assessed correlations between Ki67 distinct expressions with clinicopathology parameters. Furthermore, the prognostic significance of Ki67 in different group was also determined. Overall, we interpreted the heterogeneity of OSCC from the perspectives of Ki67 by reviewing its distinct expression and clinical studies further in searching for new successful targets for anticancer therapies.

## Materials and methods

### Patients and tissue specimens

Paraffin-embedded surgical tissues were randomly collected from 109 OSCC patients at Nanjing Stomological Hospital, Medical School, Nanjing University between 2007 and 2014. Diagnosis was confirmed by postoperative pathology and no patients received radiotherapy or chemotherapy before operation. Pregnant and patients diagnosed with other diseases were excluded from the present study. The study was carried out in accordance with the World Medical Association Declaration of Helsinki, and the approval of Ethic Committee was obtained from our hospital and informed consent from patients and their families were gained as well. All patients were followed-up until 31 July 2015.

### Reagents and antibodies

Rabbit monoclonal antibody for Ki67 (Cat No. ab15580, diluted ×200) was purchased from Abcam in U.S.A., Envision Detection System Kit used for DAB chromogen was bought from DAKO in Denmark. And xylene, ethanol, and all analytical grade solvents were obtained from Aladdin (Shanghai, China).

### Immunohistochemistry

Tissues were formalin fixed and paraffin embedded, then cut into 2 μm sections, and placed on microscope slides for immunohistochemistry. In brief, the sections were successively incubated in xylene, 100% ethanol, 95% ethanol, blocked with 3% H_2_0_2_ for 10 min at room temperature, and washed. Then all slides were incubated with a Ki67 primary antibody at 4°C overnight, followed secondary antibody, and the Envision Detection System kit was used for the DAB chromogen followed by nuclear staining using Hematoxylin.

### Immunostaining analysis

Evaluation of immunostaining was performed by two pathologists to the clinical data separately. When different opinion happened, reassessed together to reach a consensus. Cell with brown staining were considered positive under microscope. One hundred and nine patients from 2007 to 2014 were chosen to record Ki67 expression. The patterns of Ki67 expressed TCs and FLCs in OSCC tissues were counted and divided into two separated regions: CT and IF. Three representative fields in each group were picked up at 400× magnification (0.2 mm^2^/field). The numbers of Ki67 expressed TCs and FLCs were classified by ImageJ (version 1.8) and averaged for each group. The results were presented as average value per field. And optional cut-off values for Ki67 expression in TCs and FLCs at both CT and IF were confirmed according to correlations with patients’ overall survival (OS) and disease-free survival (DFS) analyzed by GraphPad Prism (version 7) [26].

### Statistical analysis

The SPSS version 16.0 (SPSS Inc., Chicago, IL, U.S.A.) and Prism statistical software package (version 7.0, GraphPad Software Inc., San Diego, CA, U.S.A.) were used for statistical analyses. The Mann–Whitney test was used to compare the two groups. OS and DFS comparing high expression groups and low expression groups were estimated using Kaplan–Meier curves. Survival time was defined as the interval between the date of surgery and the last date when the patient was known to be disease free or alive (censored). The Cox regression model was used to examine interactions between different prognostic factors in a univariate and multivariate analysis. Differences were considered statistically significant with *P*<0.05.

## Results

### Ki67 expression was much higher in TCs than in FLCs

**Figure 1:**
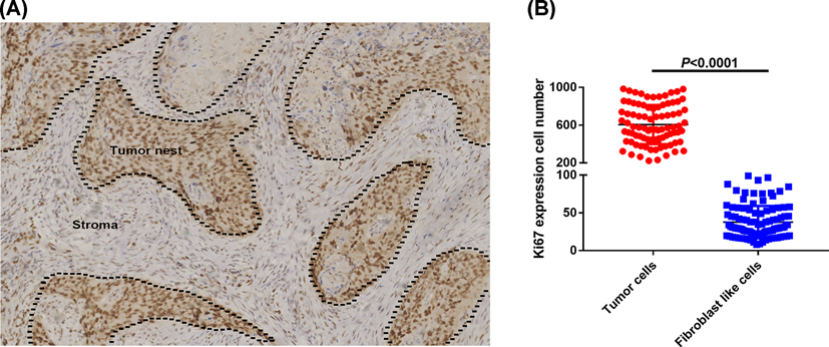
Ki67 expression in different cell types (**A**) Immunohistochemical staining showing Ki67 higher expression in TCs than FLCs; (**B**) Ki67 expression of IHC positive staining analyzed by cell numbers.

Considering the intratumor heterogeneity of malignancies, we first analyzed the expression of Ki67 in different types of cells, including TCs and non-cancer cells, such as FLCs in stroma from 109 OSCC specimens. IHC results showed Ki67 was higher expressed in TCs than FLCs in Figure 1A. The count of positively staining cells was further confirmed that compared with FLCs, TCs presented higher Ki67 expression with significant difference (Figure 1B, *P*<0.0001).

### Ki67 expression in TCs at CT was higher than IF, while Ki67 expression in FLCs at IF was higher than CT

**Figure 2:**
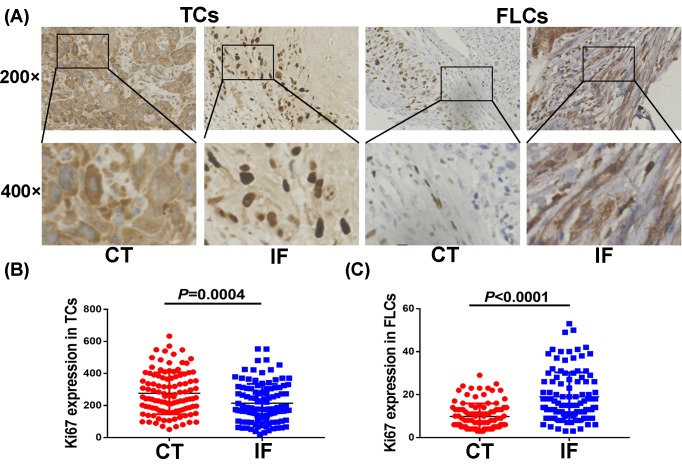
Ki67 expression in TCs and FLCs at CT and IF (**A**) IHC showed Ki67 distinct expression pattern; (**B**) Ki67 expressed in TCs at CT and IF; (**C**) Ki67 expressed in FLCs at CT and IF.

Tumor heterogeneity contains not only cell type variations but also compartment differences. Next, we divided Ki67 expression in TCs and FLCs into two different compartments including CT and IF (Figure 2A). We found that Ki67 expression in TCs was much higher at CT than that at IF (*P*=0.0004, Figure 2B). Interestingly, Ki67 expression in FLCs at CT was significantly lower than that at IF (*P*<0.0001, Figure 2C).

### Correlations between Ki67 expression in TCs and FLCs at CT and IF with their clinic pathological characteristics

To further evaluate clinical importance of Ki67 spatial expression, we analyzed correlations between Ki67 expression in TCs and FLCs at CT and IF with their clinicopathological characteristics using 109 patients’ tissues. Ki67 expression in TCs as shown in Table 1 had no significant relationships with clinicopathological characteristics neither at CT nor IF. Neither Ki67 expression in FLCs at CT did in Table 2. In addition, Ki67 expression both in CTs and FLCs at CT seemed to be a risk with gender of OSCC patients (Ki67 expression in TCs: *χ*^2^ = 1.512; Ki67 expression in FLCs: *χ*^2^ = 1.127) without significant relationships. However, higher Ki67 expression in FLCs at IF was significantly associated with lower differentiation (*P*=0.003), depth of invasion (DOI) (*P*=0.027), and worst pattern of invasion (WPOI) (*P*=0.041).

**Table 1:** Correlations between Ki67 expression in TCs at CT and IF with their clinicopathological characteristics in OSCC patients

Variable	Category	Number of cases (%)	TCs							
			Ki67 expression at CT (cutoff:252.66)				Ki67 expression at IF (cutoff:178)			
			Low	High	*χ*^2^	*P*	Low	High	*χ*^2^	*P*
Gender	Male	51 (46.8)	28 (52.8)	23 (41.1)	1.512	0.2190	29 (54.7)	22 (39.3)	0.402	0.53
	Female	58 (53.2)	25 (47.2)	33 (58.9)			24 (45.3)	34 (60.7)		
Age (years)	<60	53 (48.6)	26 (49.1)	27 (48.2)	0.008	0.93	23 (43.4)	30 (53.6)	1.129	0.288
	≥60	56 (51.4)	27 (50.9)	29 (51.8)			30 (56.6)	26 (46.4)		
TNM	I–III	109 (100)	53 (100)	56 (100)	-	-	53 (100)	56 (100)	-	-
	IV–V	0 (0)	0 (0)	0 (0)			0 (0)	0 (0)		
Differentiation	Low	56 (51.4)	32 (60.4)	24 (42.9)	3.346	0.067	27 (50.9)	29 (51.8)	0.008	0.93
	Moderate– high	53 (48.6)	21 (39.6)	32 (57.1)			26 (49.1)	27 (48.2)		
Lymph node metastasis	Yes	4 (3.7)	2 (3.8)	2 (3.6)	0.003	0.955	2 (3.8)	2 (3.6)	0.003	0.955
	No	105 (96.3)	51 (96.2)	54 (96.4)			51 (96.2)	54 (96.4)		
DOI	<4 mm	57 (52.3)	27 (50.9)	30 (53.6)	0.075	0.784	26 (49.1)	31 (55.4)	0.433	0.51
	≥4 mm	52 (47.7)	26 (49.1)	26 (46.4)			27 (50.9)	25 (44.6)		
WPOI	I–III	68 (62.4)	31 (58.5)	37 (66.1)	0.667	0.414	30 (56.6)	38 (67.9)	1.469	0.225
	IV–V	41 (37.6)	22 (41.5)	19 (33.9)			23 (43.4)	18 (32.1)		

**Table 2:** Correlations between Ki67 expression in FLCs at CT and IF with their clinicopathological characteristics in OSCC patients

Variable	Category	Number of cases (%)	FLCs							
			Ki67 expression at CT (cutoff: 11.66)				Ki67 expression at IF (cutoff: 5.33)			
			Low	High	*χ*^2^	*P*	Low	High	*χ*^2^	*P*
Gender	Male	51 (46.8)	17 (54.8)	34 (43.6)	1.127	0.288	16 (51.6)	35 (44.9)	0.405	0.5250
	Female	58 (53.2)	14 (45.2)	44 (56.4)			15 (48.4)	43 (55.1)		
Age (years)	<60	53 (48.6)	15 (48.4)	38 (48.7)	0.001	0.975	14 (45.2)	39 (50)	0.208	0.6480
	≥60	56 (51.4)	16 (51.6)	40 (51.3)			17 (54.8)	39 (50)		
TNM	I–III	109 (100)	31 (100)	78 (100)	-	-	31 (100)	78 (100)	-	-
	IV–V	0 (0)	0 (0)	0 (0)			0 (0)	0 (0)		
Differentiation	low	56 (51.4)	19 (61.3)	37 (47.4)	1.704	0.192	9 (29)	47 (60.3)	8.658	**0.003** *
	moderate– high	53 (48.6)	12 (38.7)	41 (52.6)			22 (71)	31 (39.7)		
Lymph node metastasis	Yes	4 (3.7)	1 (3.2)	3 (3.8)	0.024	0.877	1 (3.2)	3 (3.8)	0.024	0.877
	No	105 (96.3)	30 (96.8)	75 (96.2)			30 (96.8)	75 (96.2)		
DOI	<4 mm	57 (52.3)	19 (61.3)	38 (48.7)	1.406	0.236	11 (35.5)	46 (59)	4.907	**0.027** *
	≥4 mm	52 (47.7)	12 (38.7)	40 (51.3)			20 (64.5)	32 (41)		
WPOI	I–III	68 (62.4)	23 (74.2)	45 (57.7)	2.574	0.109	24 (77.4)	44 (56.4)	4.173	**0.041** *
	IV–V	41 (37.6)	8 (25.8)	33 (42.3)			7 (22.6)	34 (43.6)		

* Represented that differences were considered statistically significant with *P*<0.05.

### The prognostic significance of Ki67 in TCs and FLCs at CT and IF

**Figure 3:**
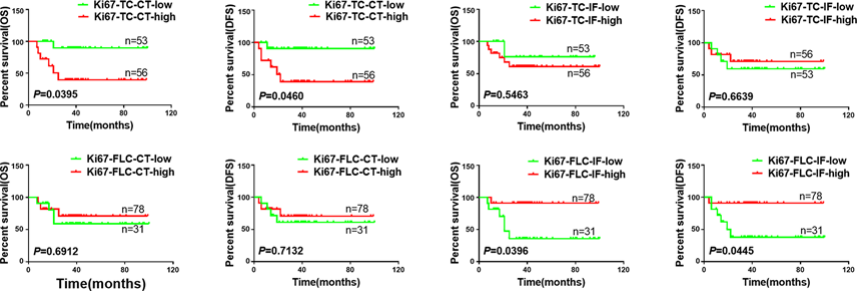
Kaplan–Meier survival curves for OS and DFS of OSCC patients according to Ki67 expression in TCs at CT (**A**,**B**) and IF (**C**,**D**), and in FLCs at CT (**E**,**F**) and IF (**G**,**H**).

The potential associations between the OS, DFS, and Ki67 spatial expression were analyzed to determine the prognostic values of Ki67 in TCs and FLCs at CT and IF. Our data demonstrated that cumulative survivals of the patients with higher Ki67 expression in TCs at CT correlated with poorer OS (*P*=0.0395, Figure 3A) and DFS (*P*=0.046, Figure 3B). However, patients with higher Ki67 expression in FLCs at IF correlated with better OS (*P*=0.0396, Figure 3G) and DFS (*P*=0.0445, Figure 3H). Additionally, there was no significant difference between OS or DFS with Ki67 expression neither in TCs at IF nor in FLCs at CT. (*P*>0.05, Figure 3C–F).

### Ki67 expression in FLC at IF was not an independent risk factor for OSCC

Univariate analyses shown in Tables 3 and 4 revealed that gender, age, TNM, differentiation, DOI were not significantly associated with OS and DFS (*P*>0.05), but lymph node metastasis (OS and DFS: *P*<0.0001), WPOI (OS and DFS: *P*=0.031), Ki67 expression in TCs at CT (OS: *P*=0.0395; DFS: *P*=0.046), and FLCs at IF (OS:*P*=0.0396; DFS:*P*=0.0445) presented to be a risk factor for OS and DFS of OSCC patients. However, multivariate analyses implicated that Ki67 expression in FLCs at IF was not an independent factor (*P*>0.05), but lymph node metastasis was an independent risk factor for OS (*P*=0.015) and DFS (*P*=0.005) in OSCC.

**Table 3:** Prognostic factors in the Cox proportional hazards model for OS

Variable	OS					
	HR	Univariate 95% CI	Sig.	HR	Multivariate 95% CI	Sig.
**Gender**						
Male compared with female	0.814	0.182–3.636	0.787	1.145	0.203–6.466	0.878
**Age (years)**						
<60 compared with ≥60	2.44	0.473–12.58	0.286	2.85	0.418–19.408	0.285
**TNM**						
III compared with IV–V	-	-	-	-	-	-
**Differentiation**						
low compared with moderate–high	2.955	0.572–15.255	0.196	0.811	0.092–7.113	0.85
**Lymph node metastasis**						
No compared with Yes	34.013	6.766–170.972	<0.0001*	11.078	1.584–77.492	0.015*
**DOI**						
<5 mm compared with ≥5 mm	69.345	0.158–30388.915	0.172	-	-	-
**WPOI**						
I–III compared with IV–V	10.281	1.237–85.407	0.031*	4.241	0.351–51.246	0.956
**Ki67 in TCs at CT**						
low compared with high	1.935	1.181–4.823	0.0395*	1.207	0.177–8.242	0.848
**Ki67 in TCs at IF**						
low compared with high	0.71	0.159–3.171	0.5463	1.462	0.271–7.885	0.658
**Ki67 in FLCs at CT**						
low compared with high	0.891	0.173–4.598	0.6912	0.101	0.008–1.208	0.07
**Ki67 in FLCs at IF**						
low compared with high	0.15	0.018–0.846	0.0396*	0.091	0.008–1.078	0.068

* Represented that differences were considered statistically significant with *P*<0.05.

**Table 4:** Prognostic factors in the Cox proportional hazards model for DFS

Variable	DFS					
	HR	Univariate 95% CI	Sig.	HR	Multivariate 95% CI	Sig.
**Gender**						
Male compared with female	0.812	0.182–3.628	0.785	0.918	0.158–5.324	0.924
**Age (years)**						
<60 compared with ≥60	2.551	0.495–13.154	0.263	4.086	0.491–33.971	0.193
**TNM**						
I–III compared with IV–V	-	-	-	-	-	-
**Differentiation**						
low compared with moderate–high	2.851	0.553–14.709	0.211	0.96	0.09–10.281	0.973
**Lymph node metastasis**						
No compared with Yes	48.626	9.378–252.131	<0.0001*	22.841	2.557–203.998	0.005*
**DOI**						
<5 mm compared with ≥5 mm	70.546	0.161–30988.958	0.17	-	-	-
**WPOI**						
I–III compared with IV–V	10.306	1.241–85.617	0.031*	4.228	0.34–52.578	0.262
**Ki67 in TCs at CT**						
low compared with high	2.974	1.189–5.023	0.046*	1.438	0.161–12.846	0.745
**Ki67 in TCs at IF**						
low compared with high	0.726	0.162–3.245	0.6639	1.916	0.295–12.458	0.496
**Ki67 in FLCs at CT**						
low compared with high	0.922	0.179–4.761	0.7132	0.119	0.01–1.479	0.098
**Ki67 in FLCs at IF**						
low compared with high	0.15	0.018–0.947	0.0445*	0.02	0.001–0.751	0.054

* Represented that differences were considered statistically significant with *P*<0.05.

## Discussion

Multiple studies have indicated advances in understanding the stromal contribution in cancer progression will improve our knowledge on the reciprocal signaling that promote cancer growth, differentiation, invasion, and survival. It is hopeful to selectively and successfully target oncogenic stromal activities beyond angiogenesis [27]. OSCC with high recurrence and metastasis remained disappointed 5-year survival after therapy, which might partly led by shortage of accurate diagnosis [19].

As a DNA-binding protein, Ki67 mainly located in the nucleus and is related to cell proliferation. With the feature of being absent in quiescent cells (G_0_) but in G_1_ phase, Ki67 has been widely used as one of the important markers in cell proliferation [28]. A number of studies have found that higher Ki67 expression is associated with poorer prognosis in breast cancer, prostate cancer, and some other cancers [29–31]. Interestingly, as an independent marker in colorectal cancers, higher Ki67 expression was found to be correlated with a favorable clinical outcome [32]. Our earlier study confirmed that Ki67 expression was also correlated with progression, and high expression of Ki67 related to poor prognosis, severe differentiation, and worse WPOI in OSCC patients. The reason for these conflicting results may partly attribute to biological features of various tumors.

Previous data revealed that the proliferative activity of a tumor is heterogeneous [33]. Tumor microenvironment containing many kinds of cells such as stromal FLCs, immune cells, and endothelial cells affects tumor progression by complicated communications with TCs [34]. Hence, it is necessary to assess Ki67 expression in regions with high proliferation, which was recommended to be IF [35]. A lower Ki67 expression at IF was associated with poor prognosis in Duke’s stage B CRC [36]. Additionally, the expression of Ki67 combined with HIF-1α and CK20 was predicted to be as prognostic biomarkers in the microenvironment of colorectal cancer tissue [37]. Therefore, it is questionable spatial Ki67 expression and its clinical prognosis.

In the present study, we uncovered Ki67 expression in different cell types (TCs and FLCs) and different compartments (tumor center and IF) and found that Ki67 expression in TCs was higher at tumor center than IF, while Ki67 expression in FLCs was higher at IF than tumor center, which predicted Ki67 might revealed distinct patterns on different compartment. Then we further investigated clinicopathologic correlations and prognosis significance of spatial Ki67 expression. The results presented that higher Ki67 expression in FLCs at IF was significantly associated with lower differentiation, DOI, WPOI, better OS, and DFS. Univariate analyses revealed Ki67 expression in FLC at IF (OS:*P*=0.0396; DFS:*P*=0.0445) to be a risk factor for OS and DFS of OSCC patients. But, multivariate analyses suggested Ki67 expression in FLC at IF could not be an independent diagnostic factor for OSCC.

In summary, the present study systematically described the use of Ki67 proliferation index to explore OSCC prognosis. Our study verified that Ki67 expression in FLCs at IF is a favor factor for OSCC progression and prognosis, these results contribute to the assumption of tumor heterogeneity amongst patients and provide potential prognostic indicator for OSCC.

## Abbreviations

CT: center of tumor
IHC: immunohistochemistry
CRC: colorectal cancer
DFS: disease-free survival
DOI: depth of invasion
FLC: fibroblast like cell
IF: invasive front
OS: overall survival
OSCC: oral squamous cell carcinoma
TC: tumor cell
WPOI: worst pattern of invasion
